# Synthesis and crystal structures of three Schiff bases derived from 3-formyl­acetyl­acetone and *o*-, *m*- and *p*-amino­benzoic acid

**DOI:** 10.1107/S2056989021013050

**Published:** 2022-01-01

**Authors:** Jan Henrik Halz, Andreas Hentsch, Christoph Wagner, Kurt Merzweiler

**Affiliations:** a Martin-Luther-Universität Halle-Wittenberg, Naturwissenschaftliche Fakultät II, Institut für Chemie, D-06099 Halle, Germany

**Keywords:** crystal structure, amino­benzoic acid, Schiff base, 3-formyl­acetyl­acetone

## Abstract

Treatment of 3-formyl­acetyl­acetone with the isomeric *o*-, *m*- and *p*-amino­benzoic acids led to the formation of the corresponding Schiff bases, namely, 3-[(2-carb­oxy­phenyl­amino)­methyl­idene]pentane-2,4-dione, **1**, 3-[(3-carb­oxy­phenyl­amino)­methyl­idene]pentane-2,4-dione, **2**, and 3-[(4-carb­oxy­phenyl­amino)­methyl­idene]pentane-2,4-dione, **3**, all C_13_H_13_NO_4_, that contain a planar amino-methyl­ene-pentane-2,4-dione core with a strong intra­molecular N—H⋯O hydrogen bridge. The carb­oxy­phenyl groups attached to the nitro­gen atom are almost coplanar to the central mol­ecular fragment. Depending on the position of the carboxyl unit, different supra­molecular structures with hydrogen-bonding networks are formed in the three title structures.

## Abstract 2

The condensation products of the *o*-, *m*- and *p*- isomers of amino­benzoic acid and 3-formyl­aceylacetone were synthesized and structurally characterized. As a result of the different substitution patterns, their crystal structures are governed by different types of hydrogen-bonding motifs.

## Chemical context

The reaction of 3-formyl­acetyl­acetone with primary amines *R*NH_2_ provides easy access to enamines with an amino-methyl­ene-pentane-2,4-dione core. This approach was used for the first time as early as 1966 by Jäger’s group in order to synthesize salen-type ligands from 3-formyl­acetyl­acetone and ethyl­enedi­amine (Wolf & Jäger, 1966 ▸). Recently, this type of ligand was applied successfully for the preparation of Fe^II^ complexes that exhibit spin-crossover effects (Dankhoff & Weber, 2019 ▸). In a previous study, we were inter­ested in the preparation of chiral *N*,*O*,*O*-ketiminate ligands from 3-formyl­acetyl­acetone and naturally occuring amino­acids (Hentsch *et al.*, 2014 ▸) and recently, we reported on *N*,*O*,*P*-ketiminates with additional PPh_2_ functionalities (Halz *et al.*, 2021 ▸). In this context, we studied the synthesis of Schiff bases derived from 3-formyl­acetyl­acetone and the isomeric *o*-, *m*- and *p*-amino­benzoic acids. The corresponding crystal structures of **1**, **2** and **3** are reported here. 
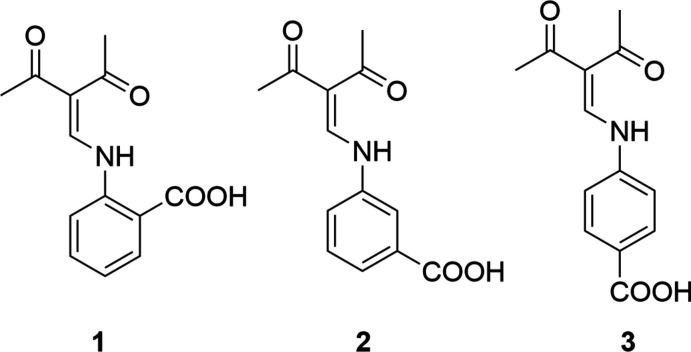


## Structural commentary

**Figure 1:**
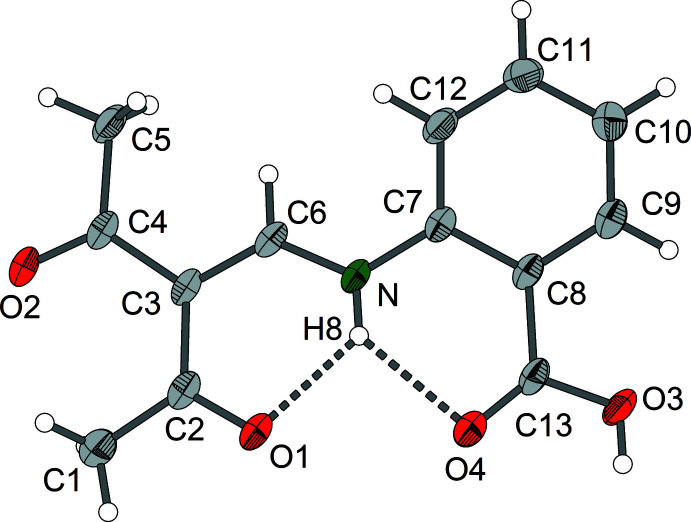
Mol­ecular structure of enamine **1** showing the labelling scheme. Hydrogen bonds are shown as dashed lines; displacement ellipsoids are drawn at the 50% probability level.

The *ortho* derivative compound **1** crystallizes in the monoclinic system, space group *C*2/*c* with *Z* = 8. Compound **2** (*meta* derivative) forms ortho­rhom­bic crystals, space group *Pnma*, *Z* = 4, and compound **3** (*para* derivative) crystallizes in the monoclinic space group *P*2_1_/*c*, *Z* = 4. Each of the three isomers **1**–**3** exists as the enamine tautomer with a central amino-methyl­ene-pentane-2,4-dione structure (Figs. 1 ▸–3 ▸ ▸). The mol­ecular structures of compounds **1** and **3** exhibit nearly planar amino-methyl­ene-pentane-2,4-dione units, and in the case of compound **2** exact planarity is observed as the mol­ecule resides on a crystallographic mirror plane perpendicular to the crystallographic *b* axis. In the case of compounds **1** and **3**, there is a small torsion of the phenyl groups [**1**: 12.16 (6)°, **3**: 30.76 (8)°] with respect to the amino-methyl­ene-pentane-2,4-dione unit.

Regarding the central amino-methyl­ene-pentane-2,4-dione part, the geometric parameters for isomers **1**–**3** are very similar (Tables 1 ▸–3 ▸ ▸). The lengths of the enamine double bonds C3=C6 range from 1.379 (2) Å in the *ortho* derivative to 1.394 (3) Å in the case of the *meta* derivative. The remaining C—C bonds at the central C3 atom are 1.443 (3)–1.482 (3) Å. In the parent compound amino-methyl­ene-pentane-2,4-dione, which may serve as a reference, the corresponding C—C distances at the central C atom are 1.397 (2) Å and 1.456 (2)–1.464 (2) Å, respectively (Gróf *et al.*, 2006 ▸). The enamine C—N bond lengths in compounds **1**–**3** are 1.333 (3)–1.337 (3) Å and thus practically identical. Generally, in this type of enamine, the C—N bond lengths for the parent amino [1.305 (2) Å] and related *N*-alkyl derivatives (*e.g*. N–CH_3_: 1.308 Å) are marginally shorter than those of *N*-aryl derivatives [*e.g*. N(*o*-NH_2_-Ph): 1.324 (2) Å] (Svensson *et al.*, 1982 ▸).

The structural differences between compounds **1**–**3** are mainly due to individual hydrogen-bonding patterns (Tables 4 ▸–6 ▸ ▸). The presence of intra­molecular N—H⋯O-type hydrogen bonds with the amine group as hydrogen donor and the acetyl oxygen atom as acceptor is typical for amino-methyl­ene-pentane-2,4-dione derivatives. However, as a result of the participation of the carboxyl groups, additional hydrogen-bonding patterns are formed.

In the case of the *ortho* derivative **1**, the intra­molecular 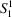 (6) type hydrogen bond between the amino group and acetyl oxygen atom O1 is extended to a bifurcated hydrogen bridge with the carbonyl oxygen atom O4 as additional acceptor. The presence of the second hydrogen bridge leads to a significant elongation of the N⋯O(acet­yl) distance [2.631 (2) Å] in comparison with the *m*- and *p*-derivatives **2** and **3** [2.598 (2) and 2.573 (2) Å, respectively].

## Supra­molecular features

For all three derivatives **1**–**3** the supra­molecular structures in the solid state are clearly governed by the presence of inter­molecular hydrogen bonds.

**Figure 4:**
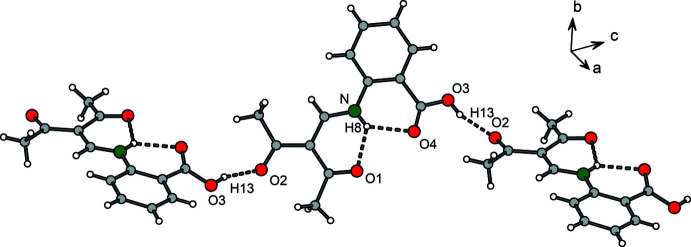
Section of the crystal structure of **1** showing the hydrogen-bonding pattern (dashed lines). Symmetry codes refer to Table 4 ▸.

**Figure 5:**
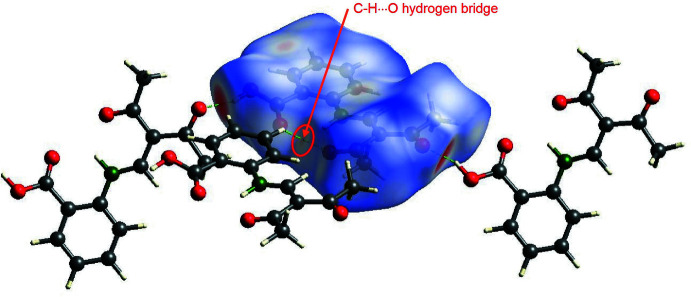
View of the Hirshfeld surface of **1** mapped over *d*_norm_ in the range −0.712 to 0.973 au showing inter­molecular hydrogen bonds as green dashed lines.

For compound **1**, the carboxyl hydrogen atom H13 forms a moderately strong hydrogen bond (Bu *et al.*, 2019 ▸; Desiraju, 2002 ▸) to the acetyl oxygen atom O2^i^ of a neighbouring mol­ecule with an O3⋯O2^i^ distance of 2.613 (2) Å (Fig. 4 ▸). The presence of this hydrogen bond is also clearly evident from the Hirshfeld surface plot (Fig. 5 ▸). Hirshfeld surface analysis (Spackman & Jayatilaka, 2009 ▸) was carried out using *CrystalExplorer* (Turner *et al.* 2017 ▸; version 17).

**Figure 6:**
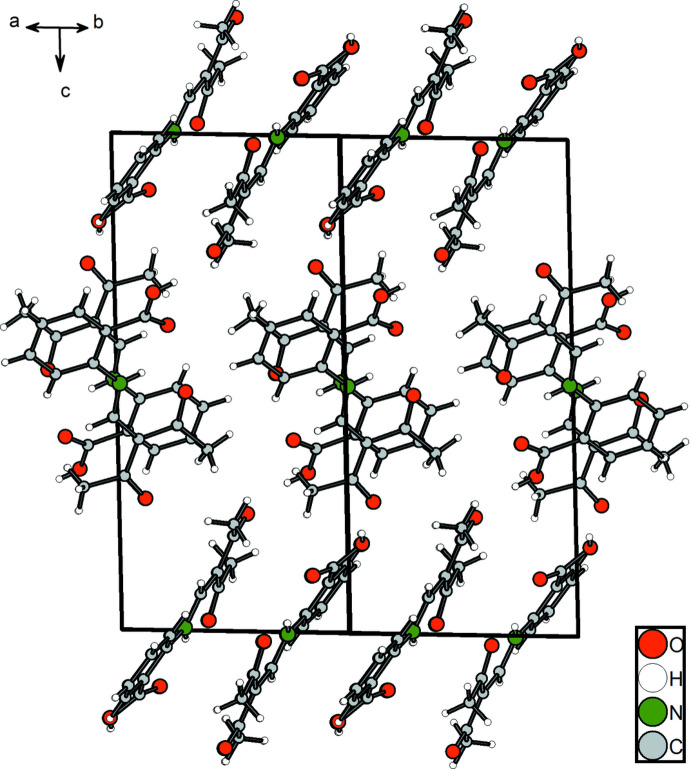
Mol­ecular packing of **1** in the crystal, in a view along [110].

As a result of these 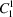 (10)-type hydrogen-bonding motifs, the Schiff base mol­ecules are linked into infinite chains propagating along [101]. One translational unit of the chain has the dimension of 20.1 Å and consists of two planar mol­ecular units, which are mutually tilted by around 51° (Fig. 6 ▸). Furthermore, the Hirshfeld surface plot hints at a weak C—H⋯O hydrogen bond between the phenyl­ene hydrogen atom H11 and the keto group oxygen atom O1^iii^ of a neighbouring chain.

**Figure 7:**
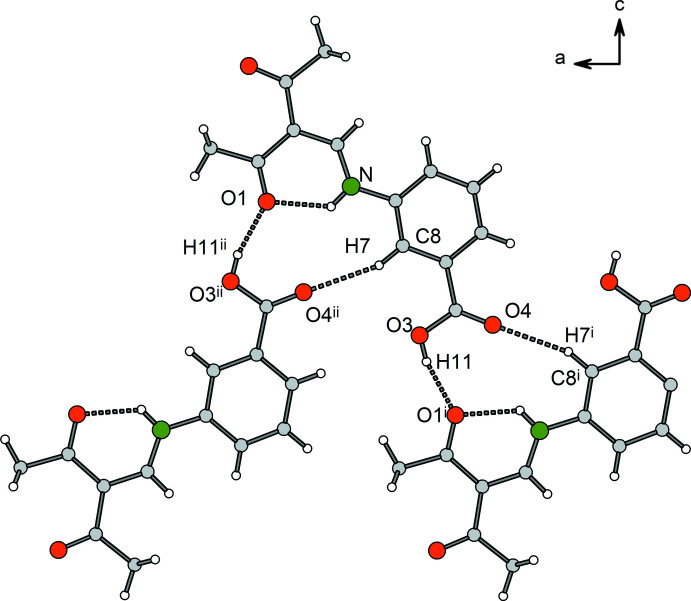
Section of the crystal structure of **2** showing the hydrogen-bonding pattern (dashed lines). Symmetry codes refer to Table 5 ▸.

**Figure 8:**
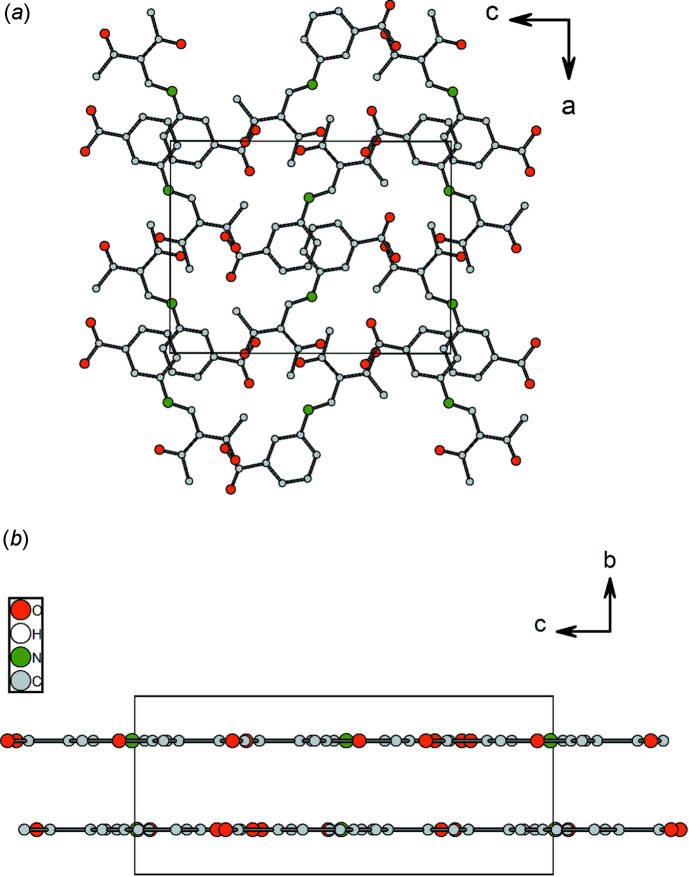
Mol­ecular packing of **2** in the crystal, (*a*) in a view along the *b* axis and (*b*) in a view along the *a* axis.

**Figure 9:**
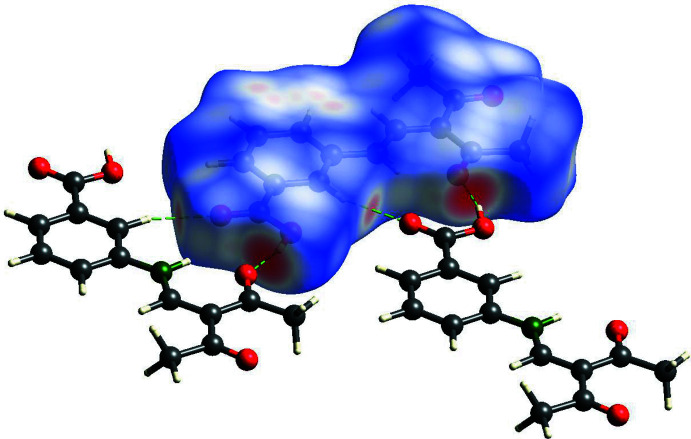
View of the Hirshfeld surface of **2** mapped over *d*_norm_ in the range −0.712 to 0.973 au showing inter­molecular hydrogen bonds as green dashed lines.

As in the case of compound **1**, the *meta* derivative **2** displays a supra­molecular chain structure. The link between the Schiff base units is provided by the hydrogen atom H11 of the carboxyl group and the acetyl oxygen atom O1^i^ of the adjacent mol­ecule with an O3⋯O1^i^ distance of 2.656 (2) Å. This connection leads to 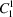 (10)-type chains in the *a-*axis direction (Fig. 7 ▸). The translational unit of the chain comprises two mol­ecular units and the repeat distance is identical to the length of the crystallographic *a* axis [11.4880 (4) Å]. In contrast to the *ortho* derivative, compound **2** exhibits exactly planar chains because of crystallographically imposed mirror symmetry (Fig. 8 ▸). Obviously, the planar arrangement is further stabilized by a weak C—H⋯O hydrogen bond between the phenyl­ene hydrogen atom H7 and the carboxyl oxygen atom O4^ii^ of an adjacent Schiff base unit, which is emphasized in the Hirshfeld surface plot (Fig. 9 ▸).

**Figure 10:**
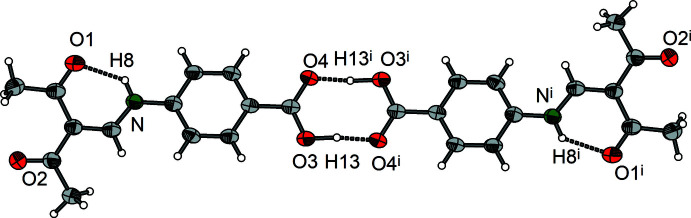
Section of the crystal structure of **3** showing the hydrogen-bonding pattern (dashed lines). Symmetry codes refer to Table 6 ▸.

**Figure 11:**
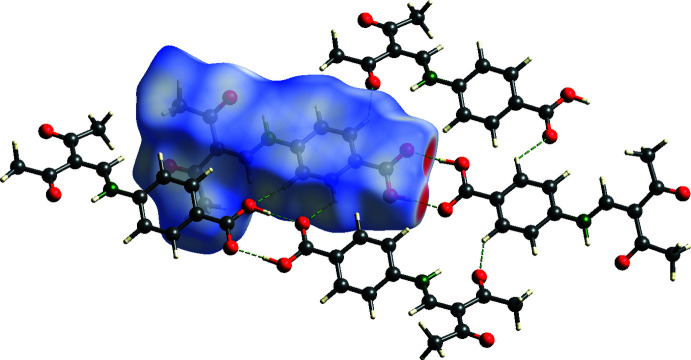
View of the Hirshfeld surface of **3** mapped over *d*_norm_ in the range −0.761 to 1.366 au showing inter­molecular hydrogen bonds as green dashed lines.

**Figure 12:**
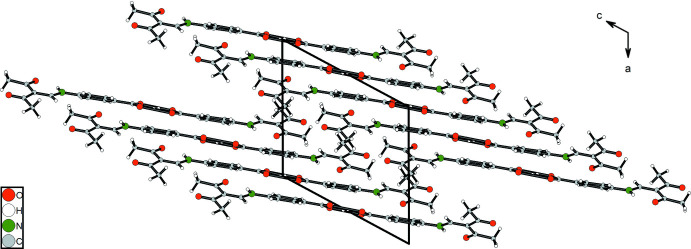
Mol­ecular packing of **3** in the crystal in a view along the *b* axis.

The *para* derivative **3** displays typical carb­oxy­lic acid dimers with an 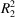 (8) motif (Fig. 10 ▸). The dimers exhibit crystallographic 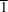 symmetry with an O3⋯O4^i^ distance of 2.6098 (18) Å that indicates a strong hydrogen bridge. Furthermore, the Hirshfeld surface plot reveals the participation of phenyl­ene hydrogen atoms in C—H⋯O hydrogen bonds (Fig. 11 ▸). Two weak C—H⋯O hydrogen bonds [C8—H8⋯O3^ii^, symmetry code: (ii) *x*, −*y* + 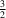 , *z* + 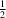 ; C9—H9⋯O4^iii^, symmetry code: (iii) −*x*, *y* + 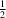 , −*z* + 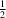 ] are formed between phenyl­ene H atoms and neighbouring carboxyl oxygen atoms, and a third inter­molecular hydrogen bond is observed between H11 and the keto group oxygen atom O1^iv^. Overall, this cross-linking leads to a layer structure that extends parallel to (100). The crystal packing is shown in Fig. 12 ▸.

## Database survey

The Cambridge Structural Database (CSD, Version 2020.3, Groom *et al.*, 2016 ▸) lists 22 Schiff base derivatives of 3-formyl­acetyl­acetone, all of which crystallize in the enamine form. Moreover, there are 19 Schiff base compounds derived from *o*-amino­benzoic acid (6 as enamine tautomers, 13 as imines), 13 from *m*-amino­benzoic acid (4 enamines, 9 imines) and 24 from *p*-amino­benzoic acid (3 enamines, 21 imines). Among the total of 53 compounds, 24 exhibit supra­molecular structures based on carb­oxy­lic acid dimers with 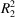 (8)-type hydrogen bridges, predominately in the case of the *m*- and *p*-amimo­benzoic acid derivatives. In the case of the *o*-amino­benzoic acid derivatives, 17 out of 19 compounds display intra­molecular N—H⋯O or O—H⋯N hydrogen bridges with an 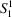 (6) topology. Additionally, there are reports on keto­imines derived from 2,4-penta­nedione and amino­benzoic acids. The corresponding *o*- and the *p*-amino­benzoic acid derivatives exist as enamines with intra­molecular N—H⋯O hydrogen bridges (Murugavel *et al.*, 2012 ▸; Joshi *et al.*, 2005 ▸). The crystal structure of the *m*-derivative has not yet been determined. Deprotonation of the amino­benzoic acid derivates was used to generate carboxyl­ates that have been applied as ligands in transition-metal complexes (Shi & Hu, 2007 ▸) and organotin compounds (Chen *et al.*, 2020 ▸; Baul *et al.*, 2008 ▸, 2009 ▸),

## Synthesis and crystallization

3-Formyl­acetyl­acetone (3.0 g, 23.4 mmol) and the corresponding amino­benzoic acid (3.3 g, 24.0 mmol) were dissolved in methanol (50 ml) and stirred at room temperature for 3 h. The solid products **1**–**3** were isolated by filtration, washed with methanol and dried *in vacuo*.

Yield: 2.7 g (47%) for **1**, 3.1 g (54%) for **2** and 3.1 g (74%) for **3** based on 3-formyl­acetyl­acetone.

Crystals suitable for single crystal X-ray diffraction of **3** were obtained from the mother liquor. In the case of compounds **1** and **2**, single crystals were obtained from a slow reaction (around three days of reaction time) of a suspension of copper(II) *o*- or *p*-amino­benzoate (1.5 g in 3 ml of water) and a solution of 3-formyl­acetyl­acetone (1.0 g in 5 ml of diethyl ether).

**1:** white powder, air stable, soluble in DMF and DMSO, hardly soluble in methanol, water, diethyl ether, THF.

C_13_H_13_NO_4_: 63.07% C (calc. 63.16%), 5.30% H (calc. 5.26%), 5.41% N (calc. 5.67%), IR: 2864 (*br*), 2586 (*w*), 1696 (*w*), 1647 (*m*), 1552 (*s*), 1492 (*m*), 1405 (*m*), 1325 (*s*), 1144 (*m*), 1077 (*w*), 978 (*m*), 935 (*m*), 789 (*m*), 759 (*s*), 695 (*m*), 652 (*m*), 634 (*s*), 584 (*s*), 544 (*m*), 470 (*m*), 405 (*m*), 326 (*m*) cm^−1^, ^1^H NMR(DMSO-*d*_6_): 2.35 (*s*, 3 H, CO—C*H*_3_), 2.39 (*s*, 3 H, CO—C*H*_3_), 7.25–7.97 (*m*, 4 H, C*H*_aromatic_), 8.39 [*d* (^3^ *J* = 12.8 Hz), 1 H, C=C*H*—-NH], 13.49 [*d* (^3^ *J* =12.8 Hz), 1 H, C=CH—N*H]*, ^13^C NMR(DMSO-*d*_6_): 27.4 ppm (–*C*H_3_), 31.4 (–C*H*_3_), 114.3 (C(O)—*C*—C(O), 117.0 (*C*H_aromatic_), 118.4 (*C*H_aromatic_), 124.0 (*C*H_aromatic_), 131.4 (*C*H_aromatic_), 134.1 (*C*H_aromatic_), 140.6 (*C*H_aromatic_), 150.6 (*C*H—NH), 167.4 (*C*OOH), 195.7 (*C*O) 198.2 (*C*O) ppm.

**2:** off-white powder, air stable, soluble in DMF and DMSO, hardly soluble in methanol, water, diethyl ether, THF.

C_13_H_13_NO_4_: 62.74% C (calc. 63.16%), 5.26% H (calc. 5.26%), 5.68% N (calc. 5.67%), IR: 2929 (*br*), 1704 (*s*), 1656 (*w*), 1632 (*s*), 1557 (*s*), 1497 (*w*), 1405 (*s*), 1347 (*m*), 1308 (*s*), 1032 (*w*), 979 (*m*), 877 (*s*), 802 (*m*), 749 (*s*), 679 (*s*), 641 (*s*), 593 (*w*), 537 (*m*), 475 (*w*), 280 (*m*), 232 (*m*) cm^−1^, ^1^H NMR(DMSO-*d*_6_): 2.37 (*s*, 3 H, CO—C*H*_3_), 2.38 (*s*, 3 H, CO–C*H*_3_), 7.52–7.94 (*m*, 4 H, C*H*_aromatic_), 8.34 [*d* (^3^ *J* = 12.8 Hz), 1 H, C=C*H*—NH], 12.53 [*d* (^3^ *J* =12.8 Hz), 1 H, C=CH—N*H*], ^13^C NMR(DMSO-*d*_6_): 27.5 ppm (–*C*H_3_), 31.4 (–C*H*_3_), 112.8 (C(O)—*C–*-C(O), 118.5 (*C*H_aromatic_), 122.5 (*C*H_aromatic_), 125.7 (*C*H_aromatic_), 129.8 (*C*H_aromatic_), 132.2 (*C*H_aromatic_), 139.4 (*C*H_aromatic_), 152.6 (*C*H—NH), 166.6 (*C*OOH), 195.2 (*C*O) 199.4 (*C*O) ppm.

**3:** yellow powder, air stable, soluble in DMF and DMSO, hardly soluble in methanol, water, diethyl ether, THF.

C_13_H_13_NO_4_: 62.79% C (calc. 63.16%), 5.27% H (calc. 5.26%), 5.53% N (calc. 5.67%), IR: 2820 (*br*), 1674 (*s*), 1628 (*s*), 1586 (*s*), 1564 (*s*), 1433 (*w*), 1390 (*s*), 1314 (*w*), 1285 (*s*), 1249 (*s*), 1206 (*m*), 1175 (*m*), 929 (*s*), 864 (*m*), 845 (*m*), 793 (*m*), 771 (*s*), 694 (*m*), 646 (*m*), 613 (*s*), 550 (*m*), 510 (*s*), 471 (*w*), 424 (*w*), 275 (*m*), 214 (*s*) cm^−1^, ^1^H NMR(DMSO-*d*_6_): 2.37 (*s*, 3 H, CO—C*H*_3_), 2.38 (*s*, 3 H, CO—C*H*_3_), 7.57 [*m*, 2 H, NHFacac-C–(C*H*_aromatic_)_2_)], 8.44 [*d* (^3^ *J* = 12.6 Hz), 1 H, C=C*H*—NH], 12.64 [*d* (^3^ *J* =12.8 Hz), 1 H, C=CH—N*H*], 12.86 (*s*, 1H, COO*H*), ^13^C NMR(DMSO-*d*_6_): 27.5 ppm (–*C*H_3_), 31.5 (–C*H*_3_), 113.4 [C(O)—*C*—C(O)], 117.7 (*C*H_aromatic_), 126.9 (*C*H_aromatic_), 130.8 (*C*H_aromatic_), 142.7 (*C*H_aromatic_), 151.7 (*C*H—NH), 166.5 (*C*OOH), 195.4 (*C*O) 199.7 (*C*O) ppm.

## Refinement

Crystal data, data collection and structure refinement details are summarized in Table 7 ▸. The methyl group hydrogen atoms of compound **2** and the carboxyl hydrogen atoms of compounds **2** and **3** were located from difference-Fourier maps and were refined freely. The remaining hydrogen atoms were positioned geometrically and refined using a riding model.

## Supplementary Material

Crystal structure: contains datablock(s) 1, 2, 3. DOI: 10.1107/S2056989021013050/wm5628sup1.cif

Structure factors: contains datablock(s) 1. DOI: 10.1107/S2056989021013050/wm56281sup5.hkl

Structure factors: contains datablock(s) 2. DOI: 10.1107/S2056989021013050/wm56282sup6.hkl

Structure factors: contains datablock(s) 3. DOI: 10.1107/S2056989021013050/wm56283sup7.hkl

Supporting information file. DOI: 10.1107/S2056989021013050/wm56281sup5.cml

Supporting information file. DOI: 10.1107/S2056989021013050/wm56282sup6.cml

Supporting information file. DOI: 10.1107/S2056989021013050/wm56283sup7.cml

CCDC references: 2127298, 2127297, 2127296

Additional supporting information:  crystallographic information; 3D view; checkCIF report

## Figures and Tables

**Figure 2:**
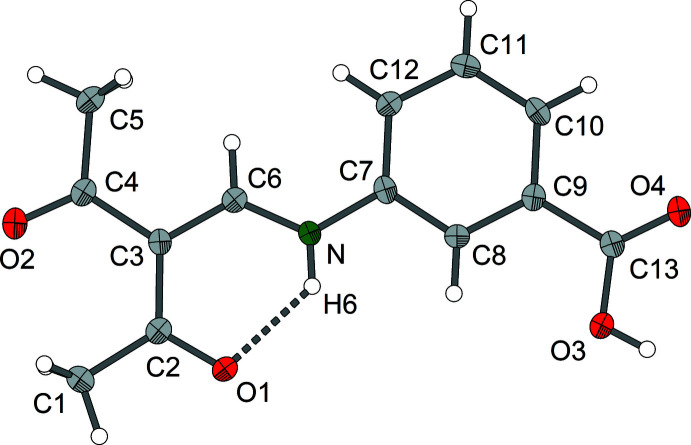
Mol­ecular structure of enamine **2** showing the labelling scheme. The hydrogen bond is shown as a dashed line; displacement ellipsoids are drawn at the 50% probability level.

**Figure 3:**
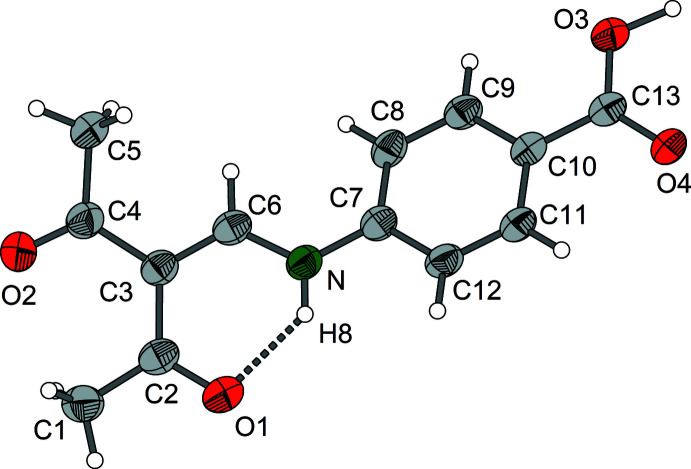
Mol­ecular structure of enamine **3** showing the labelling scheme. The hydrogen bond is shown as a dashed line; displacement ellipsoids are drawn at the 50% probability level.

**Table 1:** Selected geometric parameters (Å, °) for **1** [Chem scheme1]

O1—C2	1.2380 (18)	C2—C3	1.473 (2)
O2—C4	1.239 (2)	C3—C4	1.4621 (18)
N—C6	1.3344 (18)	C3—C6	1.383 (2)
C1—C2	1.501 (2)	C4—C5	1.513 (2)
			
C6—N—C7—C8	−167.19 (14)	C6—C3—C4—O2	176.05 (14)

**Table 2:** Selected geometric parameters (Å, °) for **2** [Chem scheme1]

O1—C2	1.243 (3)	C2—C3	1.443 (3)
O2—C4	1.226 (3)	C3—C4	1.482 (3)
N—C6	1.337 (3)	C3—C6	1.394 (3)
C1—C2	1.496 (3)	C4—C5	1.503 (3)
			
C6—N—C7—C8	180.000 (1)	C6—C3—C4—O2	180.000 (1)

**Table 3:** Selected geometric parameters (Å, °) for **3** [Chem scheme1]

O1—C2	1.2401 (19)	C2—C3	1.475 (2)
O2—C4	1.223 (2)	C3—C4	1.470 (2)
N—C6	1.333 (2)	C3—C6	1.379 (2)
C1—C2	1.487 (3)	C4—C5	1.503 (2)
			
C6—N—C7—C8	−27.4 (2)	C6—C3—C4—O2	176.88 (17)

**Table 4:** Hydrogen-bond geometry (Å, °) for **1** [Chem scheme1]

*D*—H⋯*A*	*D*—H	H⋯*A*	*D*⋯*A*	*D*—H⋯*A*
O3—H13⋯O2^i^	0.82	1.83	2.6132 (15)	160
N—H8⋯O1	0.86	2.00	2.6308 (18)	129
N—H8⋯O4	0.86	2.06	2.7266 (16)	133
C5—H6⋯O4^ii^	0.96	2.62	3.330 (2)	131
C11—H11⋯O1^iii^	0.93	2.56	3.2891 (19)	136

Symmetry codes: (i) x+{\script{1\over 2}}, -y+{\script{1\over 2}}, z+{\script{1\over 2}}; (ii) -x+{\script{1\over 2}}, -y+{\script{1\over 2}}, -z+1; (iii) x-{\script{1\over 2}}, y+{\script{1\over 2}}, z.

**Table 5:** Hydrogen-bond geometry (Å, °) for **2** [Chem scheme1]

*D*—H⋯*A*	*D*—H	H⋯*A*	*D*⋯*A*	*D*—H⋯*A*
O3—H11⋯O1^i^	0.83 (4)	1.84 (4)	2.656 (2)	166 (3)
N—H6⋯O1	0.86	1.96	2.598 (2)	130
C8—H7⋯O4^ii^	0.93	2.44	3.327 (2)	160

Symmetry codes: (i) x-{\script{1\over 2}}, y, -z+{\script{3\over 2}}; (ii) x+{\script{1\over 2}}, y, -z+{\script{3\over 2}}.

**Table 6:** Hydrogen-bond geometry (Å, °) for **3** [Chem scheme1]

*D*—H⋯*A*	*D*—H	H⋯*A*	*D*⋯*A*	*D*—H⋯*A*
O3—H13⋯O4^i^	1.12 (3)	1.49 (3)	2.6098 (18)	173 (3)
N—H8⋯O1	0.86	1.91	2.5729 (18)	133
C8—H9⋯O3^ii^	0.93	2.65	3.4832 (18)	150
C9—H10⋯O4^iii^	0.93	2.65	3.3252 (18)	130
C11—H11⋯O1^iv^	0.93	2.68	3.3612 (19)	131

Symmetry codes: (i) -x, -y+1, -z; (ii) x, -y+{\script{3\over 2}}, z+{\script{1\over 2}}; (iii) -x, y+{\script{1\over 2}}, -z+{\script{1\over 2}}; (iv) x, -y+{\script{1\over 2}}, z-{\script{1\over 2}}.

**Table 7:** Experimental details

	**1**	**2**	**3**
Crystal data			
Chemical formula	C_13_H_13_NO_4_	C_13_H_13_NO_4_	C_13_H_13_NO_4_
*M*_r_	247.24	247.24	247.24
Crystal system, space group	Monoclinic, *C*2/*c*	Orthorhombic, *P* *n* *m* *a*	Monoclinic, *P*2_1_/*c*
Temperature (K)	170	170	170
*a*, *b*, *c* (Å)	10.6287 (5), 12.3740 (4), 17.5419 (7)	11.4880 (4), 6.4726 (3), 15.2012 (5)	10.8649 (6), 10.6185 (5), 11.3616 (6)
α, β, γ (°)	90, 92.836 (3), 90	90, 90, 90	90, 118.422 (4), 90
*V* (Å^3^)	2304.28 (16)	1130.32 (8)	1152.78 (11)
*Z*	8	4	4
*D* *_x_* (Mg m^−3^)	1.425	1.453	1.425
Radiation type	Mo *K*α	Mo *K*α	Mo *K*α
μ (mm^−1^)	0.11	0.11	0.11
Crystal size (mm)	0.32 × 0.23 × 0.14	0.16 × 0.07 × 0.07	0.54 × 0.25 × 0.08

Data collection			
Diffractometer	Stoe IPDS 2T	Stoe IPDS 2T	Stoe IPDS 2T
No. of measured, independent and observed [*I* > 2σ(*I*)] reflections	5925, 2242, 2041	4667, 1346, 1118	5856, 2225, 1796
*R*_int_	0.051	0.039	0.036
(sin θ/λ)_max_ (Å^−1^)	0.617	0.639	0.617

Refinement			
*R*[*F* ^2^ > 2σ(*F* ^2^)], *wR*(*F* ^2^), *S*	0.046, 0.127, 1.06	0.044, 0.128, 1.08	0.045, 0.129, 1.06
No. of reflections	2242	1346	2225
No. of parameters	166	126	169
H-atom treatment	H-atom parameters constrained	H atoms treated by a mixture of independent and constrained refinement	H atoms treated by a mixture of independent and constrained refinement
Δρ_max_, Δρ_min_ (e Å^−3^)	0.20, −0.25	0.35, −0.25	0.27, −0.19

Computer programs: *X-AREA* (Stoe, 2016 ▸), *SHELXT* (Sheldrick, 2015*a* ▸), *SHELXL* (Sheldrick, 2015*b* ▸), *DIAMOND* (Brandenburg *et al.*, 2019 ▸) and *OLEX2* (Dolomanov *et al.*, 2009 ▸).

## supplementary crystallographic information

### 2-[(2-Acetyl-3-oxobut-1-en-1-yl)amino]benzoic acid (1) Crystal data

**Table:**

C_13_H_13_NO_4_	*F*(000) = 1040
*M**_r_* = 247.24	*D*_x_ = 1.425 Mg m^−^^3^
Monoclinic, *C*2/*c*	Mo *K*α radiation, λ = 0.71073 Å
*a* = 10.6287 (5) Å	Cell parameters from 22415 reflections
*b* = 12.3740 (4) Å	θ = 2.5–29.6°
*c* = 17.5419 (7) Å	µ = 0.11 mm^−^^1^
β = 92.836 (3)°	*T* = 170 K
*V* = 2304.28 (16) Å^3^	Block, clear colourless
*Z* = 8	0.32 × 0.23 × 0.14 mm

### 2-[(2-Acetyl-3-oxobut-1-en-1-yl)amino]benzoic acid (1) Data collection

**Table:**

Stoe IPDS 2T diffractometer	2041 reflections with *I* > 2σ(*I*)
Radiation source: sealed X-ray tube, 12 x 0.4 mm long-fine focus, Incoatec Iµs	*R*_int_ = 0.051
Plane graphite monochromator	θ_max_ = 26.0°, θ_min_ = 2.5°
Detector resolution: 6.67 pixels mm^-1^	*h* = −10→13
rotation method, ω scans	*k* = −13→15
5925 measured reflections	*l* = −21→21
2242 independent reflections	

### 2-[(2-Acetyl-3-oxobut-1-en-1-yl)amino]benzoic acid (1) Refinement

**Table:**

Refinement on *F*^2^	Primary atom site location: dual
Least-squares matrix: full	Hydrogen site location: inferred from neighbouring sites
*R*[*F*^2^ > 2σ(*F*^2^)] = 0.046	H-atom parameters constrained
*wR*(*F*^2^) = 0.127	*w* = 1/[σ^2^(*F*_o_^2^) + (0.0676*P*)^2^ + 1.813*P*] where *P* = (*F*_o_^2^ + 2*F*_c_^2^)/3
*S* = 1.06	(Δ/σ)_max_ < 0.001
2242 reflections	Δρ_max_ = 0.20 e Å^−^^3^
166 parameters	Δρ_min_ = −0.25 e Å^−^^3^
0 restraints	

### 2-[(2-Acetyl-3-oxobut-1-en-1-yl)amino]benzoic acid (1) Special details

**Table:**

Geometry. All esds (except the esd in the dihedral angle between two l.s. planes) are estimated using the full covariance matrix. The cell esds are taken into account individually in the estimation of esds in distances, angles and torsion angles; correlations between esds in cell parameters are only used when they are defined by crystal symmetry. An approximate (isotropic) treatment of cell esds is used for estimating esds involving l.s. planes.

### 2-[(2-Acetyl-3-oxobut-1-en-1-yl)amino]benzoic acid (1) Fractional atomic coordinates and isotropic or equivalent isotropic displacement parameters (Å^2^)

**Table:**

	*x*	*y*	*z*	*U*_iso_*/*U*_eq_	
O1	0.45960 (11)	0.15997 (10)	0.47933 (6)	0.0356 (3)	
O2	0.27859 (11)	0.16211 (10)	0.26411 (6)	0.0358 (3)	
O3	0.61428 (11)	0.44676 (10)	0.67913 (7)	0.0359 (3)	
H13	0.6590	0.4003	0.6999	0.054*	
O4	0.53453 (11)	0.30520 (10)	0.61407 (6)	0.0339 (3)	
N	0.36984 (11)	0.35634 (11)	0.49544 (7)	0.0239 (3)	
H8	0.4164	0.3073	0.5171	0.029*	
C1	0.45492 (18)	0.06191 (16)	0.36409 (10)	0.0401 (4)	
H1	0.5001	0.0099	0.3955	0.060*	
H2	0.5079	0.0872	0.3250	0.060*	
H3	0.3809	0.0288	0.3409	0.060*	
C2	0.41727 (14)	0.15556 (13)	0.41238 (9)	0.0285 (4)	
C3	0.33393 (13)	0.24280 (13)	0.38304 (8)	0.0245 (3)	
C4	0.26485 (13)	0.23812 (13)	0.30893 (8)	0.0259 (3)	
C5	0.17366 (15)	0.32733 (15)	0.28533 (9)	0.0320 (4)	
H4	0.1318	0.3093	0.2373	0.048*	
H5	0.2189	0.3939	0.2804	0.048*	
H6	0.1124	0.3354	0.3233	0.048*	
C6	0.31739 (13)	0.33454 (13)	0.42647 (8)	0.0245 (3)	
H7	0.2636	0.3869	0.4052	0.029*	
C7	0.35433 (13)	0.45398 (13)	0.53529 (8)	0.0232 (3)	
C8	0.43310 (13)	0.47785 (13)	0.60039 (7)	0.0233 (3)	
C9	0.41800 (14)	0.57606 (13)	0.63783 (8)	0.0277 (4)	
H9	0.4699	0.5919	0.6806	0.033*	
C10	0.32814 (15)	0.65050 (14)	0.61313 (9)	0.0303 (4)	
H10	0.3205	0.7161	0.6383	0.036*	
C11	0.24927 (15)	0.62563 (14)	0.54998 (9)	0.0316 (4)	
H11	0.1872	0.6744	0.5334	0.038*	
C12	0.26238 (14)	0.52931 (14)	0.51180 (8)	0.0297 (4)	
H12	0.2090	0.5141	0.4696	0.036*	
C13	0.53074 (14)	0.40013 (13)	0.63058 (8)	0.0250 (3)	

### 2-[(2-Acetyl-3-oxobut-1-en-1-yl)amino]benzoic acid (1) Atomic displacement parameters (Å^2^)

**Table:**

	*U*^11^	*U*^22^	*U*^33^	*U*^12^	*U*^13^	*U*^23^
O1	0.0409 (7)	0.0372 (7)	0.0267 (6)	0.0043 (5)	−0.0176 (5)	−0.0039 (5)
O2	0.0406 (7)	0.0404 (7)	0.0248 (6)	0.0031 (5)	−0.0151 (5)	−0.0082 (5)
O3	0.0385 (6)	0.0341 (7)	0.0329 (6)	−0.0001 (5)	−0.0221 (5)	−0.0002 (5)
O4	0.0402 (6)	0.0316 (7)	0.0284 (6)	0.0033 (5)	−0.0147 (5)	−0.0024 (5)
N	0.0246 (6)	0.0291 (7)	0.0173 (6)	−0.0004 (5)	−0.0070 (5)	−0.0010 (5)
C1	0.0417 (9)	0.0459 (11)	0.0315 (9)	0.0119 (8)	−0.0111 (7)	−0.0077 (8)
C2	0.0265 (7)	0.0350 (9)	0.0230 (7)	−0.0033 (6)	−0.0074 (6)	−0.0009 (6)
C3	0.0225 (7)	0.0325 (8)	0.0180 (7)	−0.0041 (6)	−0.0060 (5)	−0.0005 (6)
C4	0.0242 (7)	0.0337 (9)	0.0190 (7)	−0.0058 (6)	−0.0058 (5)	0.0006 (6)
C5	0.0305 (8)	0.0419 (10)	0.0223 (7)	0.0004 (7)	−0.0110 (6)	−0.0010 (7)
C6	0.0224 (7)	0.0329 (8)	0.0176 (7)	−0.0029 (6)	−0.0056 (5)	0.0027 (6)
C7	0.0225 (7)	0.0296 (8)	0.0171 (6)	−0.0026 (6)	−0.0026 (5)	0.0003 (6)
C8	0.0242 (7)	0.0298 (8)	0.0156 (6)	−0.0035 (6)	−0.0032 (5)	0.0016 (6)
C9	0.0298 (8)	0.0345 (9)	0.0184 (7)	−0.0037 (6)	−0.0029 (6)	−0.0009 (6)
C10	0.0329 (8)	0.0310 (8)	0.0269 (8)	−0.0002 (6)	0.0009 (6)	−0.0033 (6)
C11	0.0296 (8)	0.0342 (9)	0.0306 (8)	0.0052 (7)	−0.0030 (6)	0.0023 (7)
C12	0.0275 (7)	0.0372 (9)	0.0234 (7)	0.0014 (6)	−0.0084 (6)	0.0004 (6)
C13	0.0283 (7)	0.0314 (9)	0.0145 (6)	−0.0034 (6)	−0.0054 (5)	0.0016 (6)

### 2-[(2-Acetyl-3-oxobut-1-en-1-yl)amino]benzoic acid (1) Geometric parameters (Å, º)

**Table:**

O1—C2	1.2380 (18)	C5—H4	0.9600
O2—C4	1.239 (2)	C5—H5	0.9600
O3—H13	0.8200	C5—H6	0.9600
O3—C13	1.3312 (17)	C6—H7	0.9300
O4—C13	1.211 (2)	C7—C8	1.4135 (19)
N—H8	0.8600	C7—C12	1.398 (2)
N—C6	1.3344 (18)	C8—C9	1.394 (2)
N—C7	1.410 (2)	C8—C13	1.493 (2)
C1—H1	0.9600	C9—H9	0.9300
C1—H2	0.9600	C9—C10	1.381 (2)
C1—H3	0.9600	C10—H10	0.9300
C1—C2	1.501 (2)	C10—C11	1.390 (2)
C2—C3	1.473 (2)	C11—H11	0.9300
C3—C4	1.4621 (18)	C11—C12	1.378 (2)
C3—C6	1.383 (2)	C12—H12	0.9300
C4—C5	1.513 (2)		
			
C13—O3—H13	109.5	N—C6—C3	127.34 (14)
C6—N—H8	117.7	N—C6—H7	116.3
C6—N—C7	124.68 (13)	C3—C6—H7	116.3
C7—N—H8	117.7	N—C7—C8	120.04 (13)
H1—C1—H2	109.5	C12—C7—N	121.51 (13)
H1—C1—H3	109.5	C12—C7—C8	118.45 (14)
H2—C1—H3	109.5	C7—C8—C13	121.72 (14)
C2—C1—H1	109.5	C9—C8—C7	119.03 (14)
C2—C1—H2	109.5	C9—C8—C13	119.25 (12)
C2—C1—H3	109.5	C8—C9—H9	119.1
O1—C2—C1	118.34 (14)	C10—C9—C8	121.83 (14)
O1—C2—C3	118.90 (14)	C10—C9—H9	119.1
C3—C2—C1	122.75 (13)	C9—C10—H10	120.6
C4—C3—C2	123.26 (14)	C9—C10—C11	118.86 (15)
C6—C3—C2	119.98 (12)	C11—C10—H10	120.6
C6—C3—C4	116.76 (13)	C10—C11—H11	119.8
O2—C4—C3	121.63 (14)	C12—C11—C10	120.49 (15)
O2—C4—C5	118.30 (13)	C12—C11—H11	119.8
C3—C4—C5	120.07 (14)	C7—C12—H12	119.3
C4—C5—H4	109.5	C11—C12—C7	121.31 (14)
C4—C5—H5	109.5	C11—C12—H12	119.3
C4—C5—H6	109.5	O3—C13—C8	112.20 (13)
H4—C5—H5	109.5	O4—C13—O3	122.99 (14)
H4—C5—H6	109.5	O4—C13—C8	124.81 (13)
H5—C5—H6	109.5		
			
O1—C2—C3—C4	−171.05 (14)	C6—C3—C4—C5	−4.1 (2)
O1—C2—C3—C6	8.5 (2)	C7—N—C6—C3	176.56 (14)
N—C7—C8—C9	178.50 (13)	C7—C8—C9—C10	0.1 (2)
N—C7—C8—C13	−2.3 (2)	C7—C8—C13—O3	164.41 (13)
N—C7—C12—C11	−178.64 (14)	C7—C8—C13—O4	−16.2 (2)
C1—C2—C3—C4	9.9 (2)	C8—C7—C12—C11	1.1 (2)
C1—C2—C3—C6	−170.53 (15)	C8—C9—C10—C11	1.2 (2)
C2—C3—C4—O2	−4.4 (2)	C9—C8—C13—O3	−16.39 (19)
C2—C3—C4—C5	175.49 (14)	C9—C8—C13—O4	163.00 (15)
C2—C3—C6—N	0.1 (2)	C9—C10—C11—C12	−1.3 (2)
C4—C3—C6—N	179.70 (14)	C10—C11—C12—C7	0.2 (2)
C6—N—C7—C8	−167.19 (14)	C12—C7—C8—C9	−1.2 (2)
C6—N—C7—C12	12.5 (2)	C12—C7—C8—C13	178.00 (13)
C6—C3—C4—O2	176.05 (14)	C13—C8—C9—C10	−179.13 (14)

### 2-[(2-Acetyl-3-oxobut-1-en-1-yl)amino]benzoic acid (1) Hydrogen-bond geometry (Å, º)

**Table:**

*D*—H···*A*	*D*—H	H···*A*	*D*···*A*	*D*—H···*A*
O3—H13···O2^i^	0.82	1.83	2.6132 (15)	160
N—H8···O1	0.86	2.00	2.6308 (18)	129
N—H8···O4	0.86	2.06	2.7266 (16)	133
C5—H6···O4^ii^	0.96	2.62	3.330 (2)	131
C11—H11···O1^iii^	0.93	2.56	3.2891 (19)	136

Symmetry codes: (i) *x*+1/2, −*y*+1/2, *z*+1/2; (ii) −*x*+1/2, −*y*+1/2, −*z*+1; (iii) *x*−1/2, *y*+1/2, *z*.

### 3-[(2-Acetyl-3-oxobut-1-en-1-yl)amino]benzoic acid (2) Crystal data

**Table:**

C_13_H_13_NO_4_	*D*_x_ = 1.453 Mg m^−^^3^
*M**_r_* = 247.24	Mo *K*α radiation, λ = 0.71073 Å
Orthorhombic, *P**n**m**a*	Cell parameters from 10416 reflections
*a* = 11.4880 (4) Å	θ = 2.7–29.7°
*b* = 6.4726 (3) Å	µ = 0.11 mm^−^^1^
*c* = 15.2012 (5) Å	*T* = 170 K
*V* = 1130.32 (8) Å^3^	Needle, clear yellow
*Z* = 4	0.16 × 0.07 × 0.07 mm
*F*(000) = 520	

### 3-[(2-Acetyl-3-oxobut-1-en-1-yl)amino]benzoic acid (2) Data collection

**Table:**

Stoe IPDS 2T diffractometer	1118 reflections with *I* > 2σ(*I*)
Radiation source: sealed X-ray tube, 12 x 0.4 mm long-fine focus, Incoatec Iµs	*R*_int_ = 0.039
Plane graphite monochromator	θ_max_ = 27.0°, θ_min_ = 2.7°
Detector resolution: 6.67 pixels mm^-1^	*h* = −14→13
rotation method, ω scans	*k* = −8→7
4667 measured reflections	*l* = −18→19
1346 independent reflections	

### 3-[(2-Acetyl-3-oxobut-1-en-1-yl)amino]benzoic acid (2) Refinement

**Table:**

Refinement on *F*^2^	Primary atom site location: dual
Least-squares matrix: full	Hydrogen site location: mixed
*R*[*F*^2^ > 2σ(*F*^2^)] = 0.044	H atoms treated by a mixture of independent and constrained refinement
*wR*(*F*^2^) = 0.128	*w* = 1/[σ^2^(*F*_o_^2^) + (0.0692*P*)^2^ + 0.4804*P*] where *P* = (*F*_o_^2^ + 2*F*_c_^2^)/3
*S* = 1.08	(Δ/σ)_max_ < 0.001
1346 reflections	Δρ_max_ = 0.35 e Å^−^^3^
126 parameters	Δρ_min_ = −0.25 e Å^−^^3^
0 restraints	

### 3-[(2-Acetyl-3-oxobut-1-en-1-yl)amino]benzoic acid (2) Special details

**Table:**

Geometry. All esds (except the esd in the dihedral angle between two l.s. planes) are estimated using the full covariance matrix. The cell esds are taken into account individually in the estimation of esds in distances, angles and torsion angles; correlations between esds in cell parameters are only used when they are defined by crystal symmetry. An approximate (isotropic) treatment of cell esds is used for estimating esds involving l.s. planes.

### 3-[(2-Acetyl-3-oxobut-1-en-1-yl)amino]benzoic acid (2) Fractional atomic coordinates and isotropic or equivalent isotropic displacement parameters (Å^2^)

**Table:**

	*x*	*y*	*z*	*U*_iso_*/*U*_eq_	
O1	0.95570 (13)	0.2500	0.53720 (10)	0.0312 (4)	
O2	1.00391 (13)	0.2500	0.26645 (10)	0.0337 (4)	
O3	0.55070 (12)	0.2500	0.80351 (10)	0.0272 (4)	
H11	0.532 (3)	0.2500	0.856 (2)	0.060 (11)*	
O4	0.35794 (13)	0.2500	0.78224 (10)	0.0362 (5)	
N	0.73322 (14)	0.2500	0.50635 (11)	0.0204 (4)	
H6	0.7858	0.2500	0.5466	0.025*	
C1	1.10424 (19)	0.2500	0.43063 (16)	0.0359 (6)	
H1	1.146 (3)	0.2500	0.487 (2)	0.046 (8)*	
H2	1.1236 (17)	0.134 (3)	0.3925 (15)	0.049 (6)*	
C2	0.97887 (18)	0.2500	0.45735 (13)	0.0236 (5)	
C3	0.88505 (17)	0.2500	0.39425 (13)	0.0210 (4)	
C4	0.90572 (18)	0.2500	0.29799 (13)	0.0251 (5)	
C5	0.8029 (2)	0.2500	0.23691 (15)	0.0422 (7)	
H3	0.829 (3)	0.2500	0.175 (3)	0.069 (11)*	
H4	0.7490 (18)	0.124 (4)	0.2474 (14)	0.058 (6)*	
C6	0.76977 (17)	0.2500	0.42287 (13)	0.0209 (4)	
H5	0.7126	0.2500	0.3796	0.025*	
C7	0.61523 (17)	0.2500	0.53461 (13)	0.0190 (4)	
C8	0.59563 (16)	0.2500	0.62497 (13)	0.0195 (4)	
H7	0.6581	0.2500	0.6638	0.023*	
C9	0.48160 (17)	0.2500	0.65704 (13)	0.0206 (4)	
C10	0.38808 (17)	0.2500	0.59853 (14)	0.0225 (5)	
H8	0.3121	0.2500	0.6196	0.027*	
C11	0.40940 (17)	0.2500	0.50868 (14)	0.0238 (5)	
H9	0.3470	0.2500	0.4697	0.029*	
C12	0.52216 (18)	0.2500	0.47599 (13)	0.0213 (4)	
H10	0.5354	0.2500	0.4156	0.026*	
C13	0.45603 (17)	0.2500	0.75316 (14)	0.0232 (5)	

### 3-[(2-Acetyl-3-oxobut-1-en-1-yl)amino]benzoic acid (2) Atomic displacement parameters (Å^2^)

**Table:**

	*U*^11^	*U*^22^	*U*^33^	*U*^12^	*U*^13^	*U*^23^
O1	0.0188 (7)	0.0604 (12)	0.0144 (7)	0.000	−0.0007 (6)	0.000
O2	0.0226 (8)	0.0580 (12)	0.0205 (8)	0.000	0.0054 (6)	0.000
O3	0.0191 (8)	0.0479 (10)	0.0147 (7)	0.000	0.0015 (5)	0.000
O4	0.0184 (7)	0.0686 (13)	0.0217 (7)	0.000	0.0057 (6)	0.000
N	0.0136 (7)	0.0315 (10)	0.0162 (8)	0.000	0.0014 (6)	0.000
C1	0.0161 (10)	0.0687 (19)	0.0227 (11)	0.000	−0.0002 (8)	0.000
C2	0.0200 (9)	0.0341 (12)	0.0167 (9)	0.000	−0.0001 (8)	0.000
C3	0.0157 (8)	0.0307 (11)	0.0165 (9)	0.000	0.0020 (7)	0.000
C4	0.0221 (10)	0.0373 (12)	0.0159 (9)	0.000	0.0014 (8)	0.000
C5	0.0256 (11)	0.085 (2)	0.0156 (10)	0.000	−0.0019 (9)	0.000
C6	0.0178 (9)	0.0288 (11)	0.0160 (9)	0.000	0.0002 (7)	0.000
C7	0.0153 (9)	0.0226 (10)	0.0192 (9)	0.000	0.0032 (7)	0.000
C8	0.0156 (9)	0.0266 (10)	0.0164 (9)	0.000	−0.0009 (7)	0.000
C9	0.0180 (9)	0.0277 (11)	0.0161 (9)	0.000	0.0024 (7)	0.000
C10	0.0132 (8)	0.0307 (11)	0.0235 (10)	0.000	0.0020 (7)	0.000
C11	0.0170 (10)	0.0320 (12)	0.0225 (10)	0.000	−0.0043 (8)	0.000
C12	0.0198 (10)	0.0290 (11)	0.0151 (9)	0.000	−0.0005 (7)	0.000
C13	0.0170 (9)	0.0347 (11)	0.0178 (9)	0.000	0.0019 (7)	0.000

### 3-[(2-Acetyl-3-oxobut-1-en-1-yl)amino]benzoic acid (2) Geometric parameters (Å, º)

**Table:**

O1—C2	1.243 (3)	C5—H3	0.98 (4)
O2—C4	1.226 (3)	C5—H4	1.03 (2)
O3—H11	0.83 (4)	C6—H5	0.9300
O3—C13	1.330 (2)	C7—C8	1.392 (3)
O4—C13	1.210 (2)	C7—C12	1.392 (3)
N—H6	0.8600	C8—H7	0.9300
N—C6	1.337 (3)	C8—C9	1.398 (3)
N—C7	1.422 (2)	C9—C10	1.395 (3)
C1—H1	0.98 (3)	C9—C13	1.490 (3)
C1—H2	0.97 (2)	C10—H8	0.9300
C1—C2	1.496 (3)	C10—C11	1.388 (3)
C2—C3	1.443 (3)	C11—H9	0.9300
C3—C4	1.482 (3)	C11—C12	1.387 (3)
C3—C6	1.394 (3)	C12—H10	0.9300
C4—C5	1.503 (3)		
			
C13—O3—H11	110 (2)	C8—C7—N	116.89 (17)
C6—N—H6	117.1	C12—C7—N	122.60 (18)
C6—N—C7	125.89 (17)	C12—C7—C8	120.50 (18)
C7—N—H6	117.1	C7—C8—H7	120.1
H1—C1—H2	114.2 (15)	C7—C8—C9	119.73 (18)
C2—C1—H1	103.3 (18)	C9—C8—H7	120.1
C2—C1—H2	112.4 (12)	C8—C9—C13	121.79 (18)
O1—C2—C1	118.12 (19)	C10—C9—C8	119.96 (18)
O1—C2—C3	119.29 (19)	C10—C9—C13	118.25 (17)
C3—C2—C1	122.58 (18)	C9—C10—H8	120.3
C2—C3—C4	122.45 (17)	C11—C10—C9	119.45 (18)
C6—C3—C2	120.15 (18)	C11—C10—H8	120.3
C6—C3—C4	117.40 (18)	C10—C11—H9	119.4
O2—C4—C3	122.24 (18)	C12—C11—C10	121.16 (19)
O2—C4—C5	118.81 (19)	C12—C11—H9	119.4
C3—C4—C5	118.94 (18)	C7—C12—H10	120.4
C4—C5—H3	111 (2)	C11—C12—C7	119.20 (18)
C4—C5—H4	112.0 (12)	C11—C12—H10	120.4
H3—C5—H4	109.1 (17)	O3—C13—C9	113.76 (16)
N—C6—C3	126.50 (19)	O4—C13—O3	123.45 (19)
N—C6—H5	116.8	O4—C13—C9	122.79 (19)
C3—C6—H5	116.8		
			
O1—C2—C3—C4	180.000 (1)	C7—N—C6—C3	180.000 (1)
O1—C2—C3—C6	0.000 (1)	C7—C8—C9—C10	0.000 (1)
N—C7—C8—C9	180.000 (1)	C7—C8—C9—C13	180.000 (1)
N—C7—C12—C11	180.000 (1)	C8—C7—C12—C11	0.000 (1)
C1—C2—C3—C4	0.000 (1)	C8—C9—C10—C11	0.000 (1)
C1—C2—C3—C6	180.000 (1)	C8—C9—C13—O3	0.000 (1)
C2—C3—C4—O2	0.000 (1)	C8—C9—C13—O4	180.000 (1)
C2—C3—C4—C5	180.000 (1)	C9—C10—C11—C12	0.000 (1)
C2—C3—C6—N	0.000 (1)	C10—C9—C13—O3	180.000 (1)
C4—C3—C6—N	180.000 (1)	C10—C9—C13—O4	0.000 (1)
C6—N—C7—C8	180.000 (1)	C10—C11—C12—C7	0.000 (1)
C6—N—C7—C12	0.000 (1)	C12—C7—C8—C9	0.000 (1)
C6—C3—C4—O2	180.000 (1)	C13—C9—C10—C11	180.000 (1)
C6—C3—C4—C5	0.000 (1)		

### 3-[(2-Acetyl-3-oxobut-1-en-1-yl)amino]benzoic acid (2) Hydrogen-bond geometry (Å, º)

**Table:**

*D*—H···*A*	*D*—H	H···*A*	*D*···*A*	*D*—H···*A*
O3—H11···O1^i^	0.83 (4)	1.84 (4)	2.656 (2)	166 (3)
N—H6···O1	0.86	1.96	2.598 (2)	130
C8—H7···O4^ii^	0.93	2.44	3.327 (2)	160

Symmetry codes: (i) *x*−1/2, *y*, −*z*+3/2; (ii) *x*+1/2, *y*, −*z*+3/2.

### 4-[(2-Acetyl-3-oxobut-1-en-1-yl)amino]benzoic acid (3) Crystal data

**Table:**

C_13_H_13_NO_4_	*F*(000) = 520
*M**_r_* = 247.24	*D*_x_ = 1.425 Mg m^−^^3^
Monoclinic, *P*2_1_/*c*	Mo *K*α radiation, λ = 0.71073 Å
*a* = 10.8649 (6) Å	Cell parameters from 6441 reflections
*b* = 10.6185 (5) Å	θ = 1.9–29.6°
*c* = 11.3616 (6) Å	µ = 0.11 mm^−^^1^
β = 118.422 (4)°	*T* = 170 K
*V* = 1152.78 (11) Å^3^	Plate, clear yellowish colourless
*Z* = 4	0.54 × 0.25 × 0.08 mm

### 4-[(2-Acetyl-3-oxobut-1-en-1-yl)amino]benzoic acid (3) Data collection

**Table:**

Stoe IPDS 2T diffractometer	*R*_int_ = 0.036
Detector resolution: 6.67 pixels mm^-1^	θ_max_ = 26.0°, θ_min_ = 2.1°
rotation method, ω scans	*h* = −12→13
5856 measured reflections	*k* = −12→13
2225 independent reflections	*l* = −14→13
1796 reflections with *I* > 2σ(*I*)	

### 4-[(2-Acetyl-3-oxobut-1-en-1-yl)amino]benzoic acid (3) Refinement

**Table:**

Refinement on *F*^2^	0 restraints
Least-squares matrix: full	Hydrogen site location: mixed
*R*[*F*^2^ > 2σ(*F*^2^)] = 0.045	H atoms treated by a mixture of independent and constrained refinement
*wR*(*F*^2^) = 0.129	*w* = 1/[σ^2^(*F*_o_^2^) + (0.0728*P*)^2^ + 0.2229*P*] where *P* = (*F*_o_^2^ + 2*F*_c_^2^)/3
*S* = 1.06	(Δ/σ)_max_ < 0.001
2225 reflections	Δρ_max_ = 0.27 e Å^−^^3^
169 parameters	Δρ_min_ = −0.18 e Å^−^^3^

### 4-[(2-Acetyl-3-oxobut-1-en-1-yl)amino]benzoic acid (3) Special details

**Table:**

Geometry. All esds (except the esd in the dihedral angle between two l.s. planes) are estimated using the full covariance matrix. The cell esds are taken into account individually in the estimation of esds in distances, angles and torsion angles; correlations between esds in cell parameters are only used when they are defined by crystal symmetry. An approximate (isotropic) treatment of cell esds is used for estimating esds involving l.s. planes.

### 4-[(2-Acetyl-3-oxobut-1-en-1-yl)amino]benzoic acid (3) Fractional atomic coordinates and isotropic or equivalent isotropic displacement parameters (Å^2^)

**Table:**

	*x*	*y*	*z*	*U*_iso_*/*U*_eq_	
O1	0.26870 (14)	0.40034 (11)	0.95427 (13)	0.0496 (4)	
O2	0.47787 (18)	0.71853 (13)	1.16675 (13)	0.0659 (5)	
O3	0.05035 (12)	0.60892 (10)	0.12974 (12)	0.0429 (3)	
H13	0.010 (3)	0.600 (3)	0.019 (3)	0.132 (14)*	
O4	0.02909 (13)	0.39969 (10)	0.12604 (12)	0.0457 (3)	
N	0.25034 (14)	0.51162 (11)	0.74534 (14)	0.0372 (3)	
H8	0.2356	0.4443	0.7789	0.045*	
C1	0.3680 (2)	0.48695 (16)	1.16943 (19)	0.0471 (4)	
H3	0.4676	0.4951	1.2227	0.071*	
H1	0.3385	0.4078	1.1884	0.071*	
H2	0.3228	0.5544	1.1903	0.071*	
C2	0.32935 (16)	0.49262 (14)	1.02514 (18)	0.0392 (4)	
C3	0.36077 (16)	0.60332 (14)	0.96565 (16)	0.0366 (4)	
C4	0.44066 (17)	0.71139 (15)	1.04708 (16)	0.0418 (4)	
C5	0.47780 (19)	0.81748 (16)	0.98209 (17)	0.0455 (4)	
H6	0.5200	0.7839	0.9314	0.068*	
H4	0.5425	0.8732	1.0498	0.068*	
H5	0.3946	0.8631	0.9236	0.068*	
C6	0.31691 (16)	0.60528 (14)	0.83010 (16)	0.0370 (4)	
H7	0.3352	0.6777	0.7950	0.044*	
C7	0.20199 (15)	0.51226 (14)	0.60687 (16)	0.0350 (4)	
C8	0.16521 (16)	0.62357 (14)	0.53318 (16)	0.0379 (4)	
H9	0.1719	0.7001	0.5756	0.046*	
C9	0.11906 (16)	0.62019 (14)	0.39778 (16)	0.0372 (4)	
H10	0.0962	0.6949	0.3492	0.045*	
C10	0.10618 (15)	0.50592 (13)	0.33249 (17)	0.0335 (3)	
C11	0.14053 (17)	0.39422 (14)	0.40664 (16)	0.0378 (4)	
H11	0.1307	0.3174	0.3637	0.045*	
C12	0.18846 (17)	0.39721 (14)	0.54199 (17)	0.0392 (4)	
H12	0.2120	0.3226	0.5908	0.047*	
C13	0.05802 (15)	0.50298 (13)	0.18776 (17)	0.0360 (4)	

### 4-[(2-Acetyl-3-oxobut-1-en-1-yl)amino]benzoic acid (3) Atomic displacement parameters (Å^2^)

**Table:**

	*U*^11^	*U*^22^	*U*^33^	*U*^12^	*U*^13^	*U*^23^
O1	0.0581 (7)	0.0358 (6)	0.0532 (7)	−0.0088 (5)	0.0249 (6)	−0.0014 (5)
O2	0.0992 (11)	0.0492 (8)	0.0403 (7)	−0.0221 (7)	0.0259 (7)	−0.0058 (6)
O3	0.0523 (7)	0.0300 (6)	0.0431 (7)	−0.0037 (5)	0.0199 (5)	0.0013 (4)
O4	0.0624 (7)	0.0301 (6)	0.0440 (7)	−0.0095 (5)	0.0250 (6)	−0.0070 (4)
N	0.0399 (7)	0.0308 (7)	0.0398 (8)	−0.0021 (5)	0.0181 (6)	−0.0014 (5)
C1	0.0529 (10)	0.0405 (9)	0.0499 (10)	−0.0014 (7)	0.0262 (8)	0.0035 (7)
C2	0.0365 (8)	0.0314 (8)	0.0497 (10)	0.0023 (6)	0.0204 (7)	0.0011 (6)
C3	0.0361 (8)	0.0301 (7)	0.0421 (9)	0.0022 (6)	0.0174 (7)	0.0005 (6)
C4	0.0459 (9)	0.0336 (8)	0.0426 (9)	−0.0002 (6)	0.0184 (7)	0.0001 (6)
C5	0.0519 (10)	0.0359 (9)	0.0462 (9)	−0.0080 (7)	0.0212 (8)	−0.0034 (7)
C6	0.0358 (8)	0.0296 (7)	0.0460 (9)	0.0014 (6)	0.0197 (7)	0.0006 (6)
C7	0.0307 (7)	0.0330 (8)	0.0414 (9)	−0.0009 (6)	0.0171 (6)	−0.0015 (6)
C8	0.0397 (8)	0.0275 (7)	0.0443 (9)	0.0019 (6)	0.0182 (7)	−0.0057 (6)
C9	0.0387 (8)	0.0266 (7)	0.0446 (9)	0.0022 (6)	0.0184 (7)	0.0009 (6)
C10	0.0312 (7)	0.0279 (7)	0.0419 (9)	−0.0017 (5)	0.0177 (6)	−0.0024 (6)
C11	0.0423 (8)	0.0263 (7)	0.0441 (9)	−0.0025 (6)	0.0198 (7)	−0.0038 (6)
C12	0.0436 (8)	0.0266 (7)	0.0453 (9)	−0.0015 (6)	0.0194 (7)	0.0003 (6)
C13	0.0343 (7)	0.0283 (7)	0.0444 (9)	−0.0015 (6)	0.0180 (7)	−0.0010 (6)

### 4-[(2-Acetyl-3-oxobut-1-en-1-yl)amino]benzoic acid (3) Geometric parameters (Å, º)

**Table:**

O1—C2	1.2401 (19)	C5—H6	0.9600
O2—C4	1.223 (2)	C5—H4	0.9600
O3—H13	1.12 (3)	C5—H5	0.9600
O3—C13	1.2863 (18)	C6—H7	0.9300
O4—C13	1.2584 (18)	C7—C8	1.392 (2)
N—H8	0.8600	C7—C12	1.398 (2)
N—C6	1.333 (2)	C8—H9	0.9300
N—C7	1.402 (2)	C8—C9	1.373 (2)
C1—H3	0.9600	C9—H10	0.9300
C1—H1	0.9600	C9—C10	1.394 (2)
C1—H2	0.9600	C10—C11	1.399 (2)
C1—C2	1.487 (3)	C10—C13	1.470 (2)
C2—C3	1.475 (2)	C11—H11	0.9300
C3—C4	1.470 (2)	C11—C12	1.369 (2)
C3—C6	1.379 (2)	C12—H12	0.9300
C4—C5	1.503 (2)		
			
C13—O3—H13	113.3 (17)	N—C6—C3	125.17 (15)
C6—N—H8	116.9	N—C6—H7	117.4
C6—N—C7	126.30 (13)	C3—C6—H7	117.4
C7—N—H8	116.9	C8—C7—N	121.74 (13)
H3—C1—H1	109.5	C8—C7—C12	119.78 (15)
H3—C1—H2	109.5	C12—C7—N	118.48 (13)
H1—C1—H2	109.5	C7—C8—H9	120.0
C2—C1—H3	109.5	C9—C8—C7	119.92 (14)
C2—C1—H1	109.5	C9—C8—H9	120.0
C2—C1—H2	109.5	C8—C9—H10	119.7
O1—C2—C1	117.89 (14)	C8—C9—C10	120.63 (14)
O1—C2—C3	119.97 (16)	C10—C9—H10	119.7
C3—C2—C1	122.14 (14)	C9—C10—C11	119.16 (16)
C4—C3—C2	121.99 (15)	C9—C10—C13	120.39 (13)
C6—C3—C2	119.45 (14)	C11—C10—C13	120.45 (13)
C6—C3—C4	118.54 (14)	C10—C11—H11	119.8
O2—C4—C3	122.13 (15)	C12—C11—C10	120.44 (14)
O2—C4—C5	118.41 (15)	C12—C11—H11	119.8
C3—C4—C5	119.46 (15)	C7—C12—H12	120.0
C4—C5—H6	109.5	C11—C12—C7	120.05 (14)
C4—C5—H4	109.5	C11—C12—H12	120.0
C4—C5—H5	109.5	O3—C13—C10	117.16 (13)
H6—C5—H4	109.5	O4—C13—O3	122.59 (15)
H6—C5—H5	109.5	O4—C13—C10	120.23 (13)
H4—C5—H5	109.5		
			
O1—C2—C3—C4	−175.84 (16)	C7—N—C6—C3	178.50 (15)
O1—C2—C3—C6	2.8 (2)	C7—C8—C9—C10	1.1 (2)
N—C7—C8—C9	179.62 (14)	C8—C7—C12—C11	0.7 (2)
N—C7—C12—C11	179.51 (14)	C8—C9—C10—C11	0.2 (2)
C1—C2—C3—C4	4.2 (2)	C8—C9—C10—C13	−179.41 (14)
C1—C2—C3—C6	−177.18 (15)	C9—C10—C11—C12	−1.1 (2)
C2—C3—C4—O2	−4.5 (3)	C9—C10—C13—O3	10.8 (2)
C2—C3—C4—C5	175.83 (15)	C9—C10—C13—O4	−170.75 (15)
C2—C3—C6—N	−2.4 (2)	C10—C11—C12—C7	0.7 (3)
C4—C3—C6—N	176.29 (15)	C11—C10—C13—O3	−168.83 (14)
C6—N—C7—C8	−27.4 (2)	C11—C10—C13—O4	9.7 (2)
C6—N—C7—C12	153.77 (15)	C12—C7—C8—C9	−1.6 (2)
C6—C3—C4—O2	176.88 (17)	C13—C10—C11—C12	178.49 (15)
C6—C3—C4—C5	−2.8 (2)		

### 4-[(2-Acetyl-3-oxobut-1-en-1-yl)amino]benzoic acid (3) Hydrogen-bond geometry (Å, º)

**Table:**

*D*—H···*A*	*D*—H	H···*A*	*D*···*A*	*D*—H···*A*
O3—H13···O4^i^	1.12 (3)	1.49 (3)	2.6098 (18)	173 (3)
N—H8···O1	0.86	1.91	2.5729 (18)	133
C8—H9···O3^ii^	0.93	2.65	3.4832 (18)	150
C9—H10···O4^iii^	0.93	2.65	3.3252 (18)	130
C11—H11···O1^iv^	0.93	2.68	3.3612 (19)	131

Symmetry codes: (i) −*x*, −*y*+1, −*z*; (ii) *x*, −*y*+3/2, *z*+1/2; (iii) −*x*, *y*+1/2, −*z*+1/2; (iv) *x*, −*y*+1/2, *z*−1/2.
